# Epigenetic Regulation of Inflammatory Signaling and Inflammation-Induced Cancer

**DOI:** 10.3389/fcell.2022.931493

**Published:** 2022-06-08

**Authors:** Shawn Ying Xuan Tan, Jieqiong Zhang, Wee-Wei Tee

**Affiliations:** ^1^ Chromatin Dynamics and Disease Epigenetics Laboratory, Institute of Molecular and Cell Biology, Agency for Science, Technology and Research (A*STAR), Singapore, Singapore; ^2^ Department of Physiology, Yong Loo Lin School of Medicine, National University of Singapore, Singapore, Singapore; ^3^ NUS Centre for Cancer Research, Yong Loo Lin School of Medicine, National University of Singapore, Singapore, Singapore

**Keywords:** cancer, inflammation, epigenetics, histone modifications, high-order genome organization, super-enhancer, senescence

## Abstract

Epigenetics comprise a diverse array of reversible and dynamic modifications to the cell’s genome without implicating any DNA sequence alterations. Both the external environment surrounding the organism, as well as the internal microenvironment of cells and tissues, contribute to these epigenetic processes that play critical roles in cell fate specification and organismal development. On the other hand, dysregulation of epigenetic activities can initiate and sustain carcinogenesis, which is often augmented by inflammation. Chronic inflammation, one of the major hallmarks of cancer, stems from proinflammatory cytokines that are secreted by tumor and tumor-associated cells in the tumor microenvironment. At the same time, inflammatory signaling can establish positive and negative feedback circuits with chromatin to modulate changes in the global epigenetic landscape. In this review, we provide an in-depth discussion of the interconnected crosstalk between epigenetics and inflammation, specifically how epigenetic mechanisms at different hierarchical levels of the genome control inflammatory gene transcription, which in turn enact changes within the cell’s epigenomic profile, especially in the context of inflammation-induced cancer.

## Introduction

Chromatin structure serves as the foundation for regulating transcriptional processes, and chromatin-based alterations constitute one of the fundamental molecular mechanisms that govern cellular physiology, ranging from growth and differentiation to DNA damage repair and apoptosis. The regulation of chromatin structure via epigenetic changes, including histone modifications, chromatin remodeling and higher-order chromosomal interactions, controls the accessibility of chromatin for binding by transcription factors (TFs) and other transcriptional machinery in response to internal and external stimuli. Additionally, chromatin regulating factors interact dynamically with the epigenome to coordinate precise spatiotemporal gene expression programs that undergird cell identity and function. Misregulation of chromatin homeostasis can activate inflammatory signaling pathways that lead to the onset and development of cancer (Marazzi et al., 2018).

Inflammation is a beneficial immune defense response to curtail pathogenic infection and tissue damage. However, prolonged activation of inflammatory signaling results in chronic inflammation that can induce malignant cellular transformation. Indeed, inflammation and carcinogenesis are closely interconnected, and patients debilitated with chronic inflammatory diseases bear an increased risk of developing cancer (Garcea et al., 2005; Vagefi and Longo, 2005; Peek and Crabtree, 2006). Significant progress has emerged in recent years investigating the complex crosstalk between inflammation and tumorigenesis, switching from a cancer-centric concept to a more comprehensive view of tumor ecology that consists of epigenetically plastic cancer cells and stromal cells, which include diverse immune cells, fibroblasts and vascular cells (Greten and Grivennikov, 2019). Moreover, chronic inflammation favors a tumor-permissive microenvironment that blocks anti-tumorigenic immunity and promotes tumor development. Tumor-educated immune cells and stromal cells enable tumor immune escape and cancer progression by upregulating immune checkpoint genes and producing pathogenic immunoglobulins and cytokines (Ren et al., 2012; Simon and Labarriere, 2017; Gu et al., 2019). Therefore, immune checkpoint blockade has recently become a popular and effective form of cancer therapy.

Besides immune cells, host microbiota can contribute to a chronic inflammatory environment, which supports tumor incidence, growth and metastasis, as previously documented in gastric and colorectal cancers (Xavier et al., 2020). Interestingly, microbial organisms also act as integral components of tumor tissues in various other cancer types, such as melanoma and glioma, as well as pancreatic, breast, lung and ovarian tumors (Nejman et al., 2020). Accordingly, perturbation of tumor-resident microbiota by antibiotics elicits a predominantly inhibitory effect on breast cancer distal metastases (Fu et al., 2022). Collectively, inflammation is integral in sculpting the gene expression trajectories of stromal and cancer cells within the tumor microenvironment to favor oncogenesis, which in turn re-shapes the epigenetic landscape of immune cells and induces tumor-promoting inflammatory states to establish a positive feedback cycle for further perpetuating cancer progression.

Oncogenic and inflammatory responses are regulated by common factors and signaling pathways. A classic example is the nuclear factor kappa-light-chain-enhancer of activated B cells (NF-kB), a central transcription factor that is commonly activated in both tumor and immune cells to produce inflammatory cytokines, chemokines and growth factors, such as IL-1β, IL-6 and CCL2. Upon stimulation with the proinflammatory cues tumor necrosis factor alpha (TNFα) and lipopolysaccharide (LPS), p65, the core component of NF-kB, translocates into the nucleus, binds directly onto chromatin and induces its structural remodeling to orchestrate downstream transcriptional outputs (Brown et al., 2014). During this transactivation process, p65 also recruits and interacts with several chromatin regulators, such as epigenetic reader proteins (e.g., BRD4) and histone modifying enzymes (e.g., acetyltransferases CBP/p300) (Mukherjee et al., 2013; Hajmirza et al., 2018). Furthermore, NF-kB, in cooperation with BRD4, facilitates super-enhancer formation to trigger the production of proinflammatory transcripts (Brown et al., 2014). These observations illustrate the importance of transcription factors in directing inflammatory activation via epigenetic alterations.

In this review, we focus on the epigenetic regulation of inflammatory signaling in the context of cancer. We first describe how various chromatin modifications and histone variants function in mediating inflammatory responses. Next, we delineate the roles of chromatin structure modulation, super-enhancers and higher-order genome organization in contributing to key inflammatory transcription programs and inflammation-related oncogenic processes such as epithelial-mesenchymal transition (EMT) and senescence. Finally, we illustrate the bidirectional effects between epigenetic alterations and inflammation, as well as highlight the therapeutic application of anti-inflammatory and epigenetic drugs to combat cancer.

## Chromatin Modifications

Chromatin, a principal component of the nucleus, is organized around a fundamental repeating structure known as the nucleosome, each comprising eight core histone proteins (two each of histone H2A, H2B, H3 and H4) that scaffold the tight packaging of DNA. Protruding out of the nucleosomal structure includes the N-terminal tail of every histone and the C-terminal tail of histone H2A that permit post-translational modifications. These epigenetic changes affect chromatin structure and accessibility, thereby playing instrumental roles in regulating gene transcription in disease onset and progression, including inflammation in cancer (Bannister and Kouzarides, 2011).

Histone acetylation/deacetylation and methylation/demethylation are among the most predominant histone modifications that occur on all core histones, and they modulate inflammatory responses in both cancer and immune cells. Aside from these two histone modifications, histone phosphorylation and ubiquitination have also gradually gained attention for their crucial roles in regulating transcription and chromatin structure. As the roles of histone and DNA methylation/demethylation in cancer and inflammation have been recently and extensively reviewed (Das et al., 2021), here we focus on the mechanistic basis of histone acetylation/deacetylation, phosphorylation and ubiquitination, and how they mediate inflammatory signaling in cancer.

### Histone Acetylation and Deacetylation

Histone acetylation, one of the most prevalent histone post-translational modifications, is dynamically regulated by two protein families of opposing functions: histone acetyltransferases (HATs) and deacetylases (HDACs). HATs acetylate lysine residues of histones by transferring acetyl groups from acetyl-coenzyme A, thereby reducing the positive charge of lysine and weakening the interplay between DNA and histones (Racey and Byvoet, 1971; Bannister and Kouzarides, 2011). In contrast, HDACs remove acetyl groups from ε-N-acetyl lysine on histones (Li G. et al., 2020). The enzymatic activities of HATs and HDACs alter chromatin configuration and contribute primarily to gene activation and repression, respectively (Peserico and Simone, 2011).

HATs have been traditionally classified into two classes, type A and type B, based on their cellular localization. HAT1 (also known as KAT1), HAT2 and HAT4 constitute the solely B-type HATs, which are originally isolated from cytoplasmic extracts as they are enzymes found in the cytoplasm (Kleff et al., 1995; Parthun et al., 1996; Yang et al., 2011). They acetylate newly synthesized and free histones, particularly free histone H4, which contributes to chromatin assembly (Parthun et al., 1996; Yang et al., 2011). However, some reports have demonstrated that B-type HATs can localize to the nuclear compartment, albeit with poorly understood functions (Ruiz-Garcia et al., 1998; Ai and Parthun, 2004; Parthun, 2012). The role of B-type HATs in cancer and inflammation is also not well investigated. On the other hand, A-type HATs are a more diverse group of enzymes that predominately reside within the nucleus. According to their sequence and structure homology, A-type HATs can be further classified into three distinct families: General control non-repressible 5 (GCN5)-related N-acetyltransferases (GNATs), MYST (named after the first-identified four members MOZ, Ybf2, Sas2, and Tip60), and cAMP response element binding protein (CREB)-binding protein (CBP)/p300 proteins (Hodawadekar and Marmorstein, 2007).

The functions of A-type HATs in inflammation and cancer have been universally reported. For instance, GCN5 and its homologous partner PCAF (also known as KAT2A and KAT2B, respectively) are two well-studied GNAT family proteins, which are characterized by the presence of an acetyltransferase domain and a C-terminal bromodomain (Marmorstein, 2001). They globally acetylate core histones to upregulate gene transcription (Herrera et al., 1997; Nagy and Tora, 2007). Histone H3 lysine 9 acetylation (H3K9ac) has been highlighted as their signature target, as loss of GCN5 and PCAF in cells specifically causes H3K9ac reduction (Jin et al., 2011). Importantly, genetic deletion or pharmacological inhibition of PCAF results in a significant reduction of H3K5ac and H3K9ac levels at the promoter region of the cytokine gene IL-6, leading to its transcriptional downregulation (Xia et al., 2021). Upon treatment with the proinflammatory stimulus LPS, PCAF displays a positive correlation with H3K18ac expression, which activates the transcription levels of inflammatory genes (Huang et al., 2015). PCAF deficiency in macrophages and leukocytes leads to a remarkable decrease in the expression of inflammatory cytokines such as TNFα, CCL2 and IL-6 (de Jong et al., 2017). Additionally, degrading GCN5/PCAF by GCN5/PCAF proteolysis targeting chimera (PROTAC) downregulates inflammatory mediators in macrophages and dendritic cells (Bassi et al., 2018). Aside from histone acetylation, GCN5/PCAF can also exert non-histone acetylation functions, which play an integral role in regulating inflammation as well. For example, PCAF acetylates the KLF4 TF to facilitate its transactivation effect on IL-6 (Xia et al., 2021).

CBP/p300 proteins are conserved paralogous factors that are well known transcriptional coactivators for promoting gene transcription. Their typical substrate, histone H3 lysine 27 acetylation (H3K27ac), is widely regarded as a marker of accessible chromatin and active genes (Pasini et al., 2010; Jin et al., 2011). Inhibition of CBP/p300 has been reported to decrease H3K27ac intensity at the promoters of pivotal inflammatory response genes in macrophages, thereby regulating inflammation-related signaling networks (Peng et al., 2019). In CD4^+^ T-cells of patients suffering from the autoimmune disease systemic lupus erythematosus (SLE), CBP/p300 is recruited by the STAT family of TF proteins to confer accumulation of another active histone mark, H3K18ac, on the promoter and enhancer domains of the immunomodulatory cytokine gene IL-10, resulting in its upregulation that positively correlates with disease severity (Hedrich et al., 2014). Additionally, lower amount of H3K18ac at the promoter of another cytokine gene IL-2 in SLE patients, relative to healthy individuals, is partly attributed to the interaction between HDAC1 and CREMα (cAMP-responsive element modulator α), which contributes to histone modification changes and is induced at elevated levels in the patients’ T-cells. Similar to GCN5/PCAF, CBP/p300 can also directly interact with and acetylate non-histone proteins such as NF-κB, a key regulator of inflammatory responses (Bhatt and Ghosh, 2014). Specifically, CBP/p300 acetylates p65, a core subunit of NF-κB, at lysine 211, 218 and 310 (Chen et al., 2002). The acetylation of p65 enhances its DNA-binding ability, activates NF-κB transactivation activity and triggers expression of downstream inflammatory genes (Chen et al., 2002; Mukherjee et al., 2013).

With regard to MYST family members, Tip60 (also known as KAT5) has been shown to catalyze the deposition of H3K27ac on the promoter regions of IL-6 and IL-8 to activate pro-inflammatory signaling cascades (Wang et al., 2020). In addition, another MYST protein, MOF, which specifically acetylates histone H4 at lysine 16 (H4K16ac), regulates inflammation signaling pathways involving TNFα and IL-33 (denDekker et al., 2020; Liu et al., 2021). Taken together, type-A HATs facilitate the production of inflammatory responsive gene transcripts and modulate key mediators of inflammation by both histone and non-histone acetylation functions.

In contrast to HATs, HDACs remove acetyl groups from histones, and hence mediate histone acetylation states dynamically with HATs to regulate gene expression. Substantial evidence reveal the role of HDACs in regulating the inflammatory gene program of immune cells. For example, HDAC3 disruption causes genomic hyperacetylation, leading to the upregulation of interferon-associated genes in LPS-stimulated macrophages (Chen et al., 2012). Treatment with HDAC inhibitors (HDACi) enhances the immunomodulatory effects of T cells and natural killer (NK) cells to activate cancer immunosurveillance. A case in point is the HDACi depsipeptide (FK228) that was reported to bolster tumor antigen expression through the enrichment of H3 acetylation, which facilitates T cell cytotoxicity against melanoma (Murakami et al., 2008). Pan-HDACi, panobinostat and vorionstate, modulate the expression of the cancer-testis antigen NY-ESO-1 and enhance tumor cell recognition by NY-ESO-1-specific T-cells, thereby benefiting adoptive T cell therapy in soft tissue sarcoma (Gong et al., 2022).

NK cell-mediated tumor recognition relies on the expression of several ligands on the cell surface of tumor cells, such as UL16-binding proteins (ULBPs). Prior studies showed that HDACi treatment increases expression of ULBPs in cancer cells, which subsequently activates NK cell-mediated cytotoxicity (Lopez-Soto et al., 2009). In addition to tumor antigens, HDACi also increases the expression of NKG2D, a receptor of ULBPs and an activating cell surface receptor expressed on NK cells, triggering NK cell cytotoxic activities (Poggi et al., 2009; Yamanegi et al., 2010). Collectively, HDAC inhibition contributes to antigen processing and tumor cell recognition, which in turn activates immune cell cytotoxicity and serves as a potential pre-treatment approach for adoptive immune cell therapy to efficiently eliminate cancer cells.

### Histone Phosphorylation

Post-translational phosphorylation of histones is a fundamental epigenetic event implicated in multiple biological processes, such as DNA damage repair and carcinogenesis. It predominantly occurs in tyrosine, serine, and threonine residues on the N-terminal histone tail, which is dynamically modulated by a myriad of protein kinases and phosphatases (Nowak and Corces, 2004). In histone phosphorylation, a phosphate group from ATP is transferred to the hydroxyl group of the target amino acid, leading to a build-up of negative charge on histones, which in turn weakens histone-DNA interaction and facilitates the establishment of a transcriptionally permissive chromatin landscape (Bannister and Kouzarides, 2011).

Phosphorylation has been reported for the following histone H3 residues: serine 10, 28, threonine 3, 6, 11, 45, and tyrosine 41, as well as serine 32 of histone H2B (Shanmugam et al., 2018). Importantly, histone phosphorylation has been linked to inflammation-dependent tumorigenesis. For instance, stress-activated protein kinase 1 (MSK1) mediates phosphorylation of histone H3 at serine 10 (H3S10ph) on the promoter of NAFTC2 to activate the expression of the proinflammatory cytokines IL-6 and IL-11 in gastric cancer (Qi et al., 2020). Moreover, high levels of H3S10ph are positively associated with *Helicobacter pylori* infection-induced gastric carcinogenesis and neoplastic cellular transformation in nasopharyngeal carcinoma (Li B. et al., 2013; Yang et al., 2018). Expression of the immune regulatory cytokines IL-10 and its homolog IL-19 in macrophages is also influenced by histone H3 phosphorylation (Zhang et al., 2006), with crucial repercussions to the regulation of inflammation, as diminished expression of IL-10 and IL-19 triggers inflammatory signaling via the upregulation of inflammasome components, thereby enhancing the assembly of the inflammasome complex that promotes secretion of the proinflammatory cytokine IL-1β (Hofmann et al., 2015; Brandt et al., 2018).

Nonetheless, histone phosphorylation often does not act in isolation, but partners with other histone modifications to control gene regulatory processes. An *in vitro* study illustrated that the histone acetyltransferase GCN5 exhibits a preference for histones decorated with H3S10ph, compared to non-phosphorylated histones (Cheung et al., 2000). H3S10ph can also stabilize histone H4 acetylation, while dephosphorylation of H3S10 collaborates with HDAC1, 2 and 3-induced deacetylation of histone H4 under stress conditions (Hu et al., 2014). It has also been reported that H3S10ph assists in expanding genomic domains harboring H3K4 methylation, a marker of accessible chromatin, and restricts the propagation of heterochromatin enriched with H3K9me2 and DNA methylation (Komar and Juszczynski, 2020). Therefore, extensive crosstalk takes place between histone phosphorylation and other post-translational histone modifications to dynamically regulate gene expression patterns, especially in the context of inflammation and cancer.

### Histone Ubiquitination

Histone ubiquitination is a less well-studied post-translational modification that exerts roles in chromatin compaction and transcription regulation. It is mediated by the sequential interactions among E1, E2 and E3 enzymes: E2 is the conjugating enzyme, which transfers ubiquitin from the ubiquitin-activating enzyme E1, while E3 ligases act as protein binding platforms to catalyze the ubiquitination of substrate proteins’ lysine residues by directly transferring ubiquitin from their E2 enzymes (Berndsen and Wolberger, 2014). The function of ubiquitination primarily involves regulating the cellular localization, stability and activity of its target proteins, which include all core histone subunits. Among them, mono-ubiquitination on lysine 118 or 119 of histone H2A (H2AK118/119ub) and lysine 120 of histone H2B (H2BK120ub) are the most abundant forms of histone ubiquitination, accounting for 5–15% of H2A and 1% of H2B, respectively (Mattiroli and Penengo, 2021). H2AK118/119ub is correlated with transcriptional repression by Polycomb Repressive Complex 1 (PRC1), whereas H2BK120ub plays an important role in transcriptional elongation by the E3 enzymes RNF20 and RNF40 (Mattiroli and Penengo, 2021), both of which are associated with the DNA damage response.

H2BK120ub has been highlighted for its role in inflammation-related colorectal cancer. Specifically, the reduced levels of H2BK120ub and its E3 ligase RNF20 activate colonic inflammation and tumorigenesis by recruiting NF-kB, a master TF regulating inflammation signaling, in both mice and humans (Tarcic et al., 2016). Other studies also demonstrated that dysregulated H2BK120ub causes genomic instability, as well as promotes tumorigenesis and cancer progression in breast and lung tumors (Jeusset and McManus, 2021). Like histone phosphorylation, histone ubiquitination can also interact with and influence other histone modifications. For instance, H2BK120ub contributes to histone H3K79 and H3K4 methylation at promoter regions to induce gene transcription (Worden and Wolberger, 2019; Worden et al., 2020). Taken together, histone ubiquitination possesses roles in both transcription regulation and inflammation-induced tumorigenesis.

## Histone Variants

Further to the plethora of covalent histone modifications as described above, an under-appreciated aspect of epigenetic alteration pertinent to histones is the inclusion of non-canonical forms of these DNA-scaffolding proteins, which are commonly referred to as histone variants. Differences in these variants from the core H2A, H2B, H3 and H4 histones can be in the form of changes to the primary amino acid sequence or the incorporation of extra domains (Ghiraldini et al., 2021), thereby permitting variant-specific histone modifications that collectively influence the biochemical and physical characteristics of the nucleosome (Bonisch and Hake, 2012). For instance, even though only five amino acid residues distinguish the histone variant H3.3 from its canonical counterpart H3, euchromatic histone modifications like H3K9ac and H3K4me1 are found to accumulate selectively on H3.3 relative to H3, resulting in the elevated transcriptional activity of H3.3 enriched loci (Talbert and Henikoff, 2010).

Perhaps the most prominent biological process that showcases the increased abundance of histone variants at the expense of canonical histones is senescence, which takes place in cells undergoing irreversible proliferative arrest due to extensive stress-induced genomic damage (Hernandez-Segura et al., 2018). The accumulation of senescent cells over time triggers the inflammatory response due to the secretion of numerous signaling proteins, immune modulators, cytokines, extracellular matrix factors and proteases that make up the senescence-associated secretory phenotype (SASP) (Coppe et al., 2008; Childs et al., 2017). This in turn establishes a proinflammatory milieu that leads to chronic inflammation and induces neighboring cells to enter senescence as well, ultimately culminating in tissue dysfunction and tumorigenesis (Coppe et al., 2010; Lopez-Otin et al., 2013; Franceschi and Campisi, 2014; Lecot et al., 2016).

Examples of the loss of canonical histone proteins include the decreased expression of the core histones H3 and H4 during replicative senescence (RS) (O'Sullivan et al., 2010), which occurs in cells that experience stress induced by prolonged telomere shortening following numerous cellular divisions (Campisi and d'Adda di Fagagna, 2007). Lower levels of the linker histone H1, along with the dearth of *de novo* histone H1 synthesis from its post-translational silencing, have also been observed in cells undergoing oncogene-induced senescence (OIS) (Funayama et al., 2006), which is another type of senescence caused by induction of oncogenes and/or repression of tumor suppressor genes (Serrano et al., 1997; Sarkisian et al., 2007; Courtois-Cox et al., 2008). The reduced amount of histones adversely disrupts the global chromatin architecture, and hence exacerbates genomic damage to a greater extent (O'Sullivan et al., 2010).

On the other hand, histone variants such as histone H3.3 accumulates during cellular senescence, and its ablation resulted in cell cycle arrest *via* the repression of key cell cycle regulators (Duarte et al., 2014). Histone H2A.J, a relatively uncommon variant of H2A that exists only in mammals, is found to be enriched in DNA damage-induced senescence, and it plays a critical role in increasing the expression of inflammatory and immune-related genes during chronic inflammation, especially those implicating the SASP (Contrepois et al., 2017). Moreover, the gene encoding histone H2A.J has been documented to be aberrantly expressed in breast cancer (Colotta et al., 2009; Cornen et al., 2014; Rube et al., 2021), though its role in oncogenesis remains to be defined.

**FIGURE 1:**
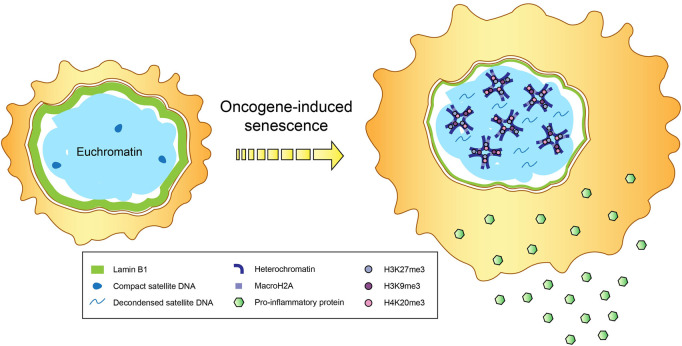
Epigenetic changes in senescence. Secretion of proinflammatory cytokines and immunomodulatory proteins that constitute the senescence-associated secretory phenotype (SASP), accompanied by the formation of senescence-associated heterochromatin domains (SAHDs) and compact senescence-associated heterochromatic foci (SAHF), occur in cells undergoing oncogene-induced senescence (OIS). In addition to the enrichment of repressive histone modifications (e.g., trimethylation of H3K9, H3K27 and H4K20) and the histone variant macroH2A within SAHFs, OIS-induced cells also tend to exhibit a reduction in lamin B1 levels and decondensation of satellite DNA, in a process called senescence-associated distension of satellites (SADS).

One of the major hallmarks of senescence is the establishment of senescence-associated heterochromatin domains (SAHDs) that are abundantly marked with H3K9me3 (Figure 1). These domains subsequently develop into senescence-associated heterochromatic foci (SAHF), which depict hotspots of compact heterochromatin decorated by a myriad of repressive epigenetic modifications like H3K27me3 and H4K20me3, and are found mainly in OIS (Narita et al., 2003; Chandra et al., 2012; Nelson et al., 2016). MacroH2A variants, the biggest known histone variants with gene repressive roles, are shown to accumulate within SAHF (Zhang et al., 2005). In particular, one of the macroH2A family isoforms, macroH2A1, is repositioned away from SASP genes to promote their expression (Chen et al., 2015), a process aided by the ATM protein kinase that is vital for regulating the cellular response to double strand breaks (DSBs), including those induced by OIS (Mallette et al., 2007). ATM also catalyzes the phosphorylation of another histone variant H2AX (commonly referred to as γH2AX) (Burma et al., 2001), which is thought to stabilize the ends of DSBs within spatial proximity for supporting DNA repair (Bassing and Alt, 2004). Notably, elevated levels of γH2AX have been documented in both cancer and inflammation-associated pathways like NF-κB signaling (Mah et al., 2010; Matsuya et al., 2022).

Nevertheless, histone variants are not always expressed at elevated levels in senescent cells. A case in point is the histone H3 variant CENP-A, which is the epigenetic marker of chromosomal centromeres that are extensively heterochromatinized and exhibit substantial changes in structure during senescence (Swanson et al., 2015). Protein levels of CENP-A are reduced in human senescent primary fibroblasts, as well as in old, compared to young, human islet cells. Accordingly, shRNA-mediated depletion of CENP-A led to premature senescence in fibroblast cells (Lee et al., 2010; Maehara et al., 2010).

## Chromatin Structure Modulation and Enhancer-Based Regulation

In order to facilitate chromatin accessibility for establishing a transcriptionally competent environment, chromatin structure can be modulated by post-translational histone alterations, such as the incorporation of methyl, acetyl or phosphate moieties, as described above. Alternatively, nucleosomes can be physically displaced by chromatin remodelers to expose the underlying genetic material for binding by RNA Polymerase II (RNAPII) and other components of the transcription machinery (Smith and Peterson, 2005).

### Chromatin Remodeling

Genes that respond to inflammatory signals can be grouped into two classes based on their requirement for chromatin remodeling: “remodeling-dependent” genes are typically characterized by the lack of promoter CpG content, with low levels of RNAPII and active histone modifications, as exemplified by the tetratricopeptide repeats-containing gene family encoding interferon-activated proteins (Ramirez-Carrozzi et al., 2009; Bhatt et al., 2012). Another example is the chromatin remodeling by oncogenic RAS of select enhancer domains that enables deposition of the active histone mark H3K27ac and recruitment of the transcriptional coactivator BRD4 *via* the pioneer TF activity of GATA4 (Nabet et al., 2015). In contrast, “remodeling-independent” genes e.g., TNF (encodes tumor necrosis factor), FOS and JUN (encode the AP1 transcription factor) often harbor RNAPII-enriched promoters with high CpG content, such that P-TEFb and other transcription elongation factors can easily bind with high accessibility for rapid gene induction (Kininis et al., 2009; Ramirez-Carrozzi et al., 2009; Xu et al., 2009).

A recent study by Alizada et al. (2021) offered key insights into the expression dynamics of both these classes of genes that are triggered by NF-kB, a master TF implicated in various inflammatory signaling pathways (Natoli, 2009). Upon its translocation into the nucleus, NF-kB binds to promoters and enhancers of proinflammatory genes to stimulate their transcription (Pierce et al., 1988). In particular, NF-kB can engage enhancers by adopting a chromatin conformation that features distal enhancer domains within three-dimensional (3D) spatial proximity to target genes (Jin et al., 2013). The most well-studied way by which NF-kB interacts with DNA is its recruitment to “remodeling-independent” genomic loci that are made transcriptionally open by the prior occupancy of other TFs (Heinz et al., 2013; Hogan et al., 2017; Link et al., 2018). These loci are often linked to the rapid expression of inflammatory genes and suppression of cell fate determination genes (Schmidt et al., 2015). Additionally, NF-kB can gain access to “remodeling-dependent” regions with the aid of transcriptional coactivators, lineage-specifying or signal-mediated TFs (Natoli, 2009; Ghisletti et al., 2010; Natoli, 2012; Freaney et al., 2013; Kaikkonen et al., 2013). Genes residing within these regions are mostly associated with dampening the inflammatory response, and they exhibit reduced activation kinetics (Natoli, 2009).

Intriguingly, NF-kB has also been demonstrated to utilize a third mode of chromatin interaction, by binding to nucleosome-occluded domains in a manner that is reminiscent of pioneer TFs, although its functional importance remains controversial (Steger and Workman, 1997; Angelov et al., 2003; Angelov et al., 2004; Lone et al., 2013; Cieslik and Bekiranov, 2015). Through comparative epigenomic investigation of the genome-wide localization dynamics of NF-kB in human, murine and bovine cells stimulated with the proinflammatory cytokine TNFα, Alizada et al. (2021) showed a substantial proportion of conserved orthologous NF-kB binding not only to accessible, but also nucleosome-bound chromatin regions. In fact, NF-kB occupancy within the latter context is likely an integral aspect of the NF-kB-induced acute inflammatory response, as reproducible results were obtained with ChIP-seq using different NF-kB subunits in diverse cell types, and these regions were significantly enriched within super-enhancer (SE) domains, which constitute about a third of all NF-kB SE binding peaks (Alizada et al., 2021).

Another notable discovery pertaining to NF-kB occupancy dynamics is that a small minority of loci with considerable NF-kB binding before TNFα treatment were the most highly expressed less than an hour after TNFα stimulation. Importantly, these NF-kB pre-bound domains were conserved across different species and cell types, harbored numerous NF-kB motifs, overlapped human non-coding inflammatory disease mutations, and belonged to several inflammation-associated SEs located in close proximity to NF-kB target genes (Alizada et al., 2021). Thus, the efficient recruitment of NF-kB to a low number of these conserved pre-bound sites bears a disproportionately robust effect on the transcriptional regulation of inflammatory genes.

The mechanistic basis of action of NF-kB involves key chromatin regulatory players like the histone acetyltransferase CBP/p300 and the epigenetic factor BRD4 (Ashburner et al., 2001; Zhong et al., 2002; Huang et al., 2009). BRD4 is part of the bromodomain and extraterminal (BET) family of transcriptional coactivators (Dey et al., 2000; LeRoy et al., 2008) that interacts with the positive transcription elongation factor P-TEFb and the SWI/SNF chromatin remodelers at active genomic loci (Jang et al., 2005; Yang et al., 2005; Shi et al., 2013). Specifically, CBP/p300 mediates NF-kB acetylation upon treatment with the proinflammatory stimuli TNFα or LPS, thereby enhancing BRD4 binding *via* its acetyl lysine-recognizing bromodomains (Greene and Chen, 2004). This interaction is essential for the productive activation of NF-kB, and heralds a key function of BRD4 in inflammatory gene transcription (Huang et al., 2009).

### Super-Enhancers

Super-enhancers (SEs) are active transcriptional hubs that consist of multiple enhancer elements densely bound by TFs and coactivators, especially the Mediator complex, and they exert crucial functions during cell fate specification and oncogenesis (Hnisz et al., 2013; Loven et al., 2013; Whyte et al., 2013). The molecular partnership between NF-kB and BRD4 is particularly evident on SE loci, where both factors are found to accumulate at significantly higher densities relative to typical enhancers and active transcription start sites. Strikingly, NF-kB cooperates with BRD4 to set up novel SE networks that govern the expression of nearby proinflammatory genes, and this is accompanied by the unexpected displacement of BRD4 from other pre-existing SE sites, such as those that regulate non-inflammatory and cell identity genes (Brown et al., 2014). These newly formed proinflammatory SEs are enriched with the p65 (canonical subunit of NF-kB) motif, indicating that direct binding of NF-kB to the new SEs is likely causal in the distribution changes of BRD4 SE occupancy in inflammation (Brown et al., 2014).

Importantly, BET bromodomain-mediated inhibition of BRD4 ablated *de novo* NF-kB-induced SE formation, which culminated in the reduction of proinflammatory gene expression, thereby illuminating the critical role of BET bromodomains in regulating global, dynamic changes in inflammatory gene transcription. Brown et al. further highlighted the physiological consequences of BRD4 inhibition *in vivo* through the disrupted responses of NF-kB-activated endothelial cells, which drive the initiation and maintenance of inflammatory phenotypes (Gimbrone et al., 1990; Ley et al., 2007), as well as the loss of inflammatory cells and atherogenesis (an inflammatory disorder) in a well-established mouse model of atherosclerosis (Brown et al., 2014).

In a separate study pertaining to SEs, Hah et al. (2015) demonstrated that following LPS treatment, upregulated genes harboring increased SE activity tend to be associated with proinflammatory transcription and immune-related processes, while downregulated genes containing decommissioned SEs are linked to chromatin organization and cell metabolism. Moreover, NF-kB and BRD4-induced SE formation is vital for proinflammatory microRNA gene activation, which is yet another epigenetic mechanism known to influence inflammation and cancer pathogenesis (Duan et al., 2016). Interestingly, inflammatory disease-specific SEs can be further differentiated from the archetypal NF-kB-mediated SEs. For instance, the RUNX1 and ETS1 TFs showed elevated binding levels within SE loci of synovial-fluid derived CD4 T lymphocytes in patients with the autoimmune disorder juvenile idiopathic arthritis (JIA), leading to a greater expression of inflammatory genes regulated by these JIA-associated SEs including interleukins and chemokine receptors (Peeters et al., 2015). Collectively, these findings reveal SEs as potential therapeutic targets for controlling inflammation and immune-related gene regulatory networks by perturbing inflammatory SE architecture and function.

From an evolutionary standpoint, the origin of numerous enhancers can be traced back to endogenous retroviruses (ERVs), such that gene regulatory programs driving inflammatory phenotypes have gradually gained enhancer elements by co-opting genomic sequences from ERVs (Chuong et al., 2013; Chuong et al., 2016). Additionally, enhancer-encoded RNA and its chromatin milieu often undergo post-translational alterations (Li et al., 2016). Therefore, certain enhancers are able to establish a specific epigenetic memory of the initial inflammatory signal in a phenomenon called enhancer bookmarking, which contributes to innate “trained” immunity and promotes a quicker response to future stimulatory cues (Ostuni et al., 2013).

## Higher-Order Spatial Genome Organization

Beyond the epigenetic regulation of inflammatory gene transcription by histone modifications, chromatin remodeling and SE dynamics, as discussed in the previous sections, higher-order genome topology of varying hierarchical levels, ranging from long range chromatin looping within the same and across different chromosomes to topologically associating domains (TADs) that make up A (euchromatin) and B (heterochromatin) compartments, also undergird the multi-faceted nature of chromatin-dependent inflammatory responses. A case in point is highlighted by the increased appreciation of promoters from different genes aggregating in close spatial proximity to facilitate their co-regulation (Li et al., 2012), to the extent that some promoters appear to possess enhancer capabilities, dubbed “ePromoters,” which were found to come together in 3D space to regulate the interferon-α response (Dao et al., 2017).

### Transcription Factories and Chromatin Loops

The advent of chromosome conformation capture (3C) techniques led to the understanding that transcription regulation is not confined to a linear segment of chromatin, but occurs within defined nuclear regions called transcription factories, in which RNAPII and members of the transcriptional apparatus that are far apart in 3D space can colocalize with one another during gene activation (Dekker et al., 2002; Osborne et al., 2004; Papantonis et al., 2010; Larkin et al., 2012; Papantonis et al., 2012; Sharaf et al., 2014). Inflammatory genes are generally not found in transcription factories prior to stimulation, but swiftly localize to these specialized domains upon activation by proinflammatory signals (Papantonis et al., 2010; Larkin et al., 2012; Papantonis et al., 2012). For example, LPS treatment resulted in the close spatial assembly of the regulatory elements of IL-1A, IL-1B and IL-37 cytokine genes in human monocytes, suggesting co-regulation within a specific transcription factory (Sharaf et al., 2014). Papantonis et al. (2012) uncovered the crucial role of active NF-kB-mediated transcription factories in coordinating select nascent mRNA and non-coding miRNA production, following TNFα-induced stimulation.

Notably, transcriptional dynamics within transcription factories operate in a hierarchical fashion involving both cis and trans chromosomal interactions (Fanucchi et al., 2013). Such changes in chromatin spatial configurations have been elegantly illustrated in the context of antigen stimulation of naïve T lymphocytes, which differentiate into Th1, Th2 and Th17 cells that express distinct cytokine genes located on different chromosomes. The Th2 cytokine locus is instrumental for establishing long-range chromatin contacts with three promoters that regulate the genes specifying IL-4, IL-5 and IL-13 interleukins across hundreds of kilobases on the same chromosome (Spilianakis and Flavell, 2004; Lee et al., 2005). Additionally, this highly accessible Th2 locus can associate with the IL-17 and IFN-γ gene promoters located on different chromosomes. Intriguingly, such inter-chromosomal crosstalk is abrogated in favor of intra-chromosomal interactions upon cytokine gene activation, which is a unique approach harnessed by naïve T cells to alter its developmental trajectory for counter-balancing chronic inflammation (Spilianakis et al., 2005; Kim et al., 2014).

NF-kB, the master regulator of multiple inflammatory signaling pathways, also leverages on higher-order genome organization to discharge its gene regulatory roles (Kolovos et al., 2016). For instance, activation of NF-kB upon a viral infection provokes long range chromatin re-wiring between the IFN-β gene locus and three distant NF-kB bound loci on separate chromosomes, which is characterized by a diminution of these inter-chromosomal contacts at the onset of transcriptional initiation and elongation, relative to its inactive state (Apostolou and Thanos, 2008). In another study, NF-kB occupancy on the microRNA gene loci of miR-155 and miR-146a, located on different chromosomes, led to their colocalization and concomitant gene suppression during the induction of endotoxin tolerance in activated naïve macrophages (Doxaki et al., 2015).

Calandrelli et al. (2020) recently dissected the global changes in 3D spatial chromatin dynamics in stress-induced transcriptional dysregulation of endothelial cells, which feature prominently in several diseases. Treatment with TNFα and high glucose levels that mimic the inflammatory response in diabetic patients not only resulted in the loss of the repressive histone modifications H3K9me3 and H3K27me3, thereby activating inflammatory NF-kB target genes, but also significantly enhanced genome-wide inter-chromosomal RNA-chromatin interactions, particularly at sites harboring super-enhancer loci that drive proinflammatory gene expression and endothelial-mesenchymal transition (Calandrelli et al., 2020).

CTCF, a well-known architectural insulator protein that plays integral roles in both intra- and inter-chromosomal genome organization (Ong and Corces, 2014), has also been implicated in the inflammatory response modulation by TNFα and LPS stimuli. For example, treatment with TNFα induced the formation of enhancer-promoter loops at the human cytokine genes lymphotoxin-α (LTα) and TNFα, as well as the promoter region of another NF-kB-responsive gene LTβ, but loss of CTCF diminished TNF expression while promoting LTβ activation (Watanabe et al., 2012). Nikolic et al. (2014) also reported a drastic decrease in the production of TNFα and the IL-10 family of cytokines in activated macrophages lacking CTCF. LPS treatment was found to trigger CTCF detachment, accompanied by non-coding RNA expression at the chicken lysozyme genomic locus in macrophages (Lefevre et al., 2008; Witham et al., 2013).

### Topologically Associating Domains (TADs) and A/B Compartments

The classic role of CTCF in regulating 3D genome architecture is attributed to its insulator function at the boundary between TADs, which are sub-megabase chromatin regions that can self-associate by forming loops with cis-regulatory elements and their target genes within the domain, while restricting interactions outside the domain (Dixon et al., 2012; Dixon et al., 2016). At the next genomic layer, chromatin is broadly partitioned to two large-scale compartments: transcriptionally open euchromatic (A) versus compact heterochromatic (B) compartments (Kempfer and Pombo, 2020). Inflammatory challenges can impinge on 3D chromatin topology at both the TAD and A/B compartment levels, thereby altering gene expression profiles and cell fates, as discussed in this section.

One of the essential processes to quell inflammation is the production of IL-4 cytokines, which induce macrophage polarization to the anti-inflammatory M2 population (Mills et al., 2000). Phanstiel et al. (2017) uncovered distinct differences in the chromatin landscape of naïve macrophages before IL-4 stimulation, compared to those treated with IL-4 and then rested for a day. In addition, differentiation of human monocytes to macrophages initiates spatial chromatin modifications at the TAD level, with enrichment of the stress-associated and cell type-specific TF AP-1 on active enhancer-bound loops at key macrophage genes, as opposed to undifferentiated monocytes (Phanstiel et al., 2017).

Viruses have been demonstrated to hijack and re-wire the 3D chromatin organization of the host cell for subverting its immune defense system and exerting long-term inflammatory and other gene regulatory effects (Heinz et al., 2018; Liu et al., 2020). In light of the ongoing COVID-19 pandemic, Wang et al. (2021) recently reported that SARS-CoV-2 infected cells showed a significant ablation of cohesin, another architectural protein complex that collaborates with CTCF to mediate DNA looping (Dekker and Mirny, 2016), within TADs, causing a widespread weakening of intra-TAD chromatin interactions. Furthermore, A/B compartmentalization manifested a drastic perturbation in the form of A-to-B switching, resulting in erosion of the euchromatic A compartment that is coupled with a global decrease in the active histone H3K27ac mark. The physiological ramifications of these epigenetic disruptions and higher-order chromatin reconfigurations included downregulation of antiviral interferon response genes and upregulation of proinflammatory genes, shedding important insights into the inflammatory phenotypes observed in COVID-19 patients (Carvalho et al., 2021).

Importantly, 3D genome organization is a key driver of cellular senescence, which enacts chromatin restructuring at multiple levels, ranging from an increase in local chromatin interactions to a global shortening of chromosomal arms (Criscione et al., 2016). Zirkel et al. (2018) revealed one example of such chromatin reconfiguration stemming from the loss of HMGB2 at several TAD borders in senescent cells. HMGB2 belongs to the family of high-mobility group (HMG) proteins, which are ubiquitous non-histone regulatory factors that bind to and influence chromatin architecture (Reeves, 2001; Bianchi and Agresti, 2005). Senescence-mediated abolishment of HMGB2 led to the anomalous assembly of CTCF clusters, and in line with the insulating function of HMGB2, novel long range CTCF-based loops were established at genomic sites where HMGB2 previously occupied (Zirkel et al., 2018).

Another recent report addressed chromatin looping aberrations during OIS. Specifically, enhancer-promoter contacts at the IL-1 cytokine gene cluster, where key cell cycle and SASP-related genes reside, were disrupted, resulting in the increased expression of proinflammatory genes and silencing of cell cycle genes (Olan et al., 2020). These alterations are partially due to the transcription-mediated redistribution of cohesin, forming “cohesin islands” that arise from the accumulation of the cohesin complex at the 3’ ends of active genes caused by the inefficient removal of cohesin, which in turn generates new cohesin-induced DNA loops (Busslinger et al., 2017). Nonetheless, TAD boundaries and A/B compartmentalization remain largely unaffected in OIS (Chandra et al., 2015; Olan et al., 2020).

Both OIS and RS forms of senescence exhibit a dampening of short-range chromatin contacts, but an increase in long range genomic interactions (Sati et al., 2020). Moreover, A/B compartment transitions are highly conserved in both types of senescence, which correspond to downstream transcriptional outcomes in the form of gene activation for B-to-A compartment switches and gene repression for A-to-B compartment changes. However, A/B compartmentalization differences are also evident, as OIS features elevated B-B and diminished A-B interactions, while RS displays diminished A-A and elevated A-B interactions (Sati et al., 2020). Importantly, the architectural protein condensin plays a critical role in sustaining the senescent phenotype, as it functions in B-to-A compartment switching and stabilizes the A compartment, thereby enabling senescence-associated gene induction (Iwasaki et al., 2019). Additionally, genes within the vicinity of SAHF are expectedly downregulated (Iwasaki et al., 2019), yet Sati et al. (2020) reported that SAHF can serve as hubs for the aggregation of select gene loci to facilitate their expression, especially genes pertaining to inflammation and oncogenesis.

OIS is widely believed to hinder oncogenesis, owing to its role in restraining cellular proliferation, but it can also promote cancer development through the effect of certain SASP molecules on the cells’ immune system, such as the recruitment of anti-inflammatory M2 macrophages by CCL2 cytokines that sets up an immunosuppressive environment for supporting cancer progression (Allavena et al., 2008), as well as the secretion of proinflammatory SASP factors IL-6 and IL-8 by senescent fibroblasts that stimulates prostate cancer development in mice (Laberge et al., 2015). Interestingly, in colorectal cancer, Johnstone et al. (2020) recently highlighted a weakening of A/B compartmentalization, along with the establishment of a novel intermediate compartment that features long range chromatin interactions with both A and B compartments. However, the silencing histone H3K27me3 modification is found to accumulate in this intermediate compartment specifically in tumor cells, accompanied by the repression of genes residing within it, yet some genes encoding cancer-testis antigens (CTAs) and ERVs become unexpectedly upregulated (Johnstone et al., 2020), a phenomenon that has previously been observed in colon tumors and associated with pro-immunity and viral mimicry roles (Rooney et al., 2015; Roulois et al., 2015; Gibbs and Whitehurst, 2018).

### Nuclear Substructures and Chromosome Territories

On top of genomic macro-domains like TADs and A/B compartments, heterochromatinization engenders the 3D nuclear structure (Falk et al., 2019), which consists of regions associated with the nucleolus and nuclear lamina, including pericentric heterochromatin (Guenatri et al., 2004; Nemeth et al., 2010; van Steensel and Belmont, 2017). Chromatin localization to various substructures within the nucleus is important for regulating its transcriptional status, as active genes tend to be found within the nuclear interior and/or in proximity to nuclear speckles that abound with splicing factors (Lamond and Spector, 2003; Kim et al., 2020), while inactive genes typically border the lamina in regions termed as lamina-associated domains (LADs) and/or nucleolar peripheries (Nemeth et al., 2010; Kind et al., 2015; van Steensel and Belmont, 2017). Finally, individual chromosomes are preferentially arranged within defined areas of the nuclear space to form chromosome territories that represent the apex global level of chromatin organization (Meaburn and Misteli, 2007; Fritz et al., 2019).

At the level of LADs, cells undergoing OIS exhibited a heterochromatic lamina-specific reduction of chromatin contacts, whereby these GC-poor domains were transcriptionally closed and adorned with H3K9me3 (Chandra et al., 2015). Despite the loss of LAD-mediated interactions, these regions could still coalesce in spatial proximity with one another that is reminiscent of SAHF establishment (Chandra et al., 2015). Analysis by polymer modeling lent further support to the roles of LAD detachment and SAHD decompaction in the development of OIS-induced SAHF (Sati et al., 2020). A different study using senescent human lung fibroblasts illustrated the physical condensation of individual chromosomes that accounts for the generation of SAHF (Funayama et al., 2006). Nonetheless, even though SAHF domains are replete with repressive proteins and histone modifications, they are not found within constitutive heterochromatic domains like centromeres and telomeres (Narita et al., 2003; Funayama et al., 2006; Zhang et al., 2007). There is hitherto no report involving alterations to chromosome territories as a result of inflammatory signaling or inflammation-induced senescence.

## Repetitive Elements

A central epigenetic theme in cellular senescence is the genome-wide chromatin remodeling of repetitive sequences, which encompass up to two-thirds of the entire human genome (de Koning et al., 2011). This is usually manifested in the transcriptional relaxation of transposable elements such as Alu, SINE-VNTR-Alus and LINE-1, thereby facilitating non-coding RNA (ncRNA) expression from these loci and their mobilization, which activates several inflammatory/immunological gene networks including the cGAS-STING signaling pathway, type-1 interferon (IFN-1) response and the SASP (De Cecco et al., 2013; Criscione et al., 2016; De Cecco et al., 2019). Specifically, silencing of retrotransposons is performed by multiple heterochromatic players like HP1, DNMT1 and SIRT6. Hence, cells lacking the SIRT6 histone deacetylase showed an increase in LINE-1 transcripts that induced a robust IFN-1 output by activating cGAS (Simon et al., 2019).

De Cecco et al. (2019) recently delineated the mechanistic basis underpinning the aberrant activation of LINE-1 retrotransposons during senescence, which entailed depletion of the RB1 tumor suppressor protein by relieving the silencing histone H3K9me3 and H3K27me3 marks [RB1 has been reported to occupy LINE-1 and other repetitive loci to aid in their repression (Ishak et al., 2016)], increased binding of the pioneer TF FOXA1 to the LINE-1 promoter region for its activation [senescent cells show upregulation of FOXA1 (Li Q. et al., 2013)], and loss of the 3’ exonuclease TREX1 that removes foreign invading DNA species (Thomas et al., 2017), causing the accumulation of LINE-1 cDNA (De Cecco et al., 2019). Despite the delayed onset of LINE-1 reactivation and its accompanying IFN-1 response, they are crucial contributors to the proinflammatory outcome and maturation to the full-fledged SASP, including the expression of key cytokines IL-6, CCL2 and MMP3. Notably, the establishment of innate immune signatures following senescence-mediated LINE-1 induction takes place *via* the interferon-stimulatory DNA route, and treatment with nucleoside reverse transcriptase inhibitors (NRTIs) that target the reverse transcriptase function of LINE-1 (Dai et al., 2011) can significantly ameliorate both the IFN-1 response and chronic inflammation in diverse tissue types (De Cecco et al., 2019).

In another study, mouse embryonic fibroblasts transfected with LINE-1 expression plasmids demonstrated a heightened IFN-β immune response that requires the ORF2 endonuclease function of LINE-1, implying the necessity of LINE-1’s transposase activity in IFN-β activation (Yu et al., 2015). Interestingly, the interplay between LINE-1 and IFN-β sets up a negative feedback loop, as exogenous or induced IFN-β can in turn hinder LINE-1 transposition (Yu et al., 2015).

Besides transposable elements, the deleterious reconfiguration and reactivation of repetitive elements in senescent cells can also affect non-mobile centromeric and satellite DNA, leading to substantial structural changes in a process called senescence-associated distension of satellites (SADS), during which these typically constitutively silenced genomic sequences become decondensed and gain transcriptional accessibility (Swanson et al., 2013; Criscione et al., 2016) (Figure 1). These elements are also hypomethylated, in line with their distension and derepression (Cruickshanks et al., 2013). The occurrence of SADS precedes SAHF formation, and marks one of the initial alterations to the epigenetic landscape in cellular senescence (Swanson et al., 2013; Criscione et al., 2016), but the requirement of SADS in triggering and/or sustaining the senescent state remains unknown. Importantly, the loss of linker histone H1, which is a common chromatin modification observed during senescence (Funayama et al., 2006), is not causal of SADS, as most SADS-containing cells still possess high amounts of H1 protein (Swanson et al., 2013). Swanson et al. (2013) postulated that SADS may instead be attributed to the depletion of lamin B1, as almost all cells harboring normal endogenous levels of lamin B1 maintained compact heterochromatinized satellite sequences, compared to about a quarter of cells with decreased lamin B1 showcasing satellite distension.

In a similar vein, human lung fibroblasts exposed to X-ray-induced senescence elicited a dramatic increase in ncRNA expression from pericentromeric repetitive loci known as human satellite II (hSATII), which are usually repressed in healthy cells (Miyata et al., 2021). Mechanistically, these chromatin-associated hSATII RNA bind and sequester CTCF, which in turn impedes CTCF function by changing its genomic occupancy and rewiring 3D chromatin conformation particularly at SASP gene loci, leading to an increase in chromatin accessibility of these genes’ regulatory elements that induces SASP proinflammatory gene transcription (Miyata et al., 2021). In fact, lower levels of CTCF in aged cells can promote pericentromeric satellite RNA transcription and further abrogate CTCF function through a positive feedback cycle, which consequently enhances SASP-mediated inflammation and oncogenesis during the aging process (Miyata et al., 2021). This may partly explain the appearance of transformed foci in embryonic fibroblast-derived cells of CTCF-haploinsufficient (Ctcf+/-) mice, which become exceptionally prone to developing cancer (Kemp et al., 2014), and Ctcf-null mice are inviable beyond early embryogenesis (Moore et al., 2012).

## Inflammation-Induced Epigenetic Alterations

**FIGURE 2:**
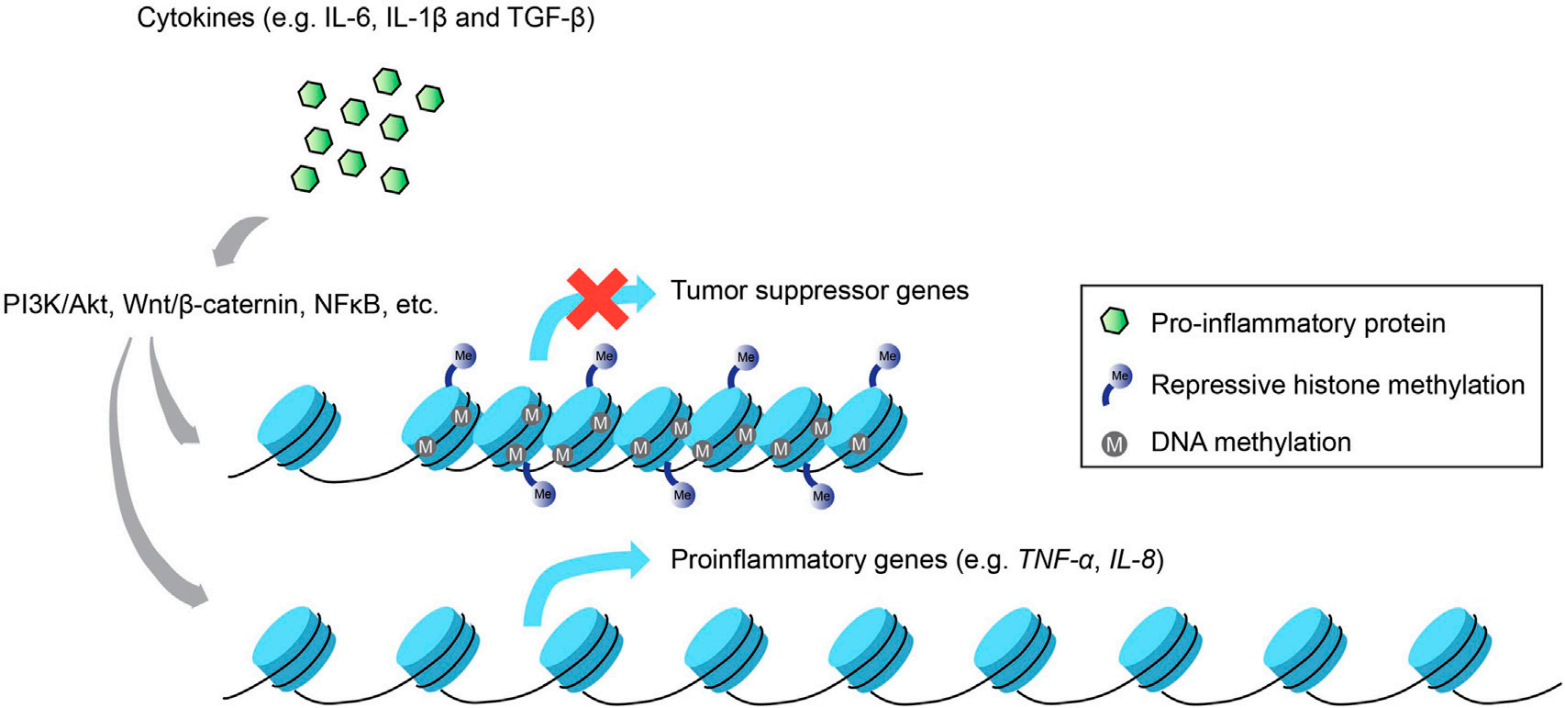
Inflammation-induced epigenetic alterations. Not only do epigenetic modifications regulate inflammatory gene expression, the activation of inflammatory signaling pathways via proinflammatory cytokines can also enact changes to the epigenetic landscape that result in the silencing of tumor suppressor genes and the increased activation of proinflammatory genes that promote carcinogenesis.

Thus far, we have described how epigenetic changes at different hierarchical levels of the eukaryotic genome regulate the expression of inflammatory and immunological genes, translating to downstream physiological consequences that control cell function and disease state. Nevertheless, the reverse relationship, i.e., how inflammatory signals impinge on the chromatin landscape, also bears significant relevance to fully appreciate the crosstalk that exists between these two molecular entities, especially in the context of cancer (Figure 2).

One of the most well-studied diseases associated with chronic inflammation that subsequently re-wires the host epigenome is gastric cancer caused by the bacterium *Helicobacter pylori*, which activates proinflammatory gene transcription via multiple signaling pathways such as PI3K/Akt, Wnt/β-catenin and NF-kB (Yamaoka et al., 2004; Lu et al., 2005; Tabassam et al., 2009). Inflammation-induced epigenetic perturbations that ensued from gastric mucosa cells infected by *H. pylori* included upregulation of proinflammatory genes, e.g., TNFα and IL-1β caused by aberrant modifications in DNA methylation of their promoter regions (Maeda et al., 2017). These alterations are believed to be linked to infection-induced inflammation and not the infection per se, since methylome changes directly influenced the expression profiles of various inflammation-associated genes in a gerbil model, and treatment with an immunosuppressant drug mitigated these methylation changes with negligible effects on bacterial colonization (Kurkjian et al., 2008; Katayama et al., 2009; Niwa et al., 2010). Furthermore, inflammation-induced DNA methylation dysregulation precipitated by infection with *H. pylori* or Epstein-Barr virus in the gastric mucosa drives gene expression changes that bolster oncogenesis, including tumor suppressor genes like LOX and p16Ink4a, and proinflammatory genes like IL-8 and TNFα (Matsusaka et al., 2014).

Katayama et al. (2009) reported that the DNA methylation alterations were largely attributed to macrophage production of nitric oxide in response to *H. pylori* infection. In cervical cancer, nitric oxide-induced inflammation is also culpable for affecting the promoter methylation levels of multiple genes, including cancer-related genes, e.g., protein tyrosine phosphatase receptor type R (PTPRR), and genes with immune functions, e.g., T-lymphocyte maturation-associated protein (MAL) (Su et al., 2017; Holubekova et al., 2020), thereby establishing the causal connection between infection-driven inflammatory signaling and its downstream epigenetic changes.

Inflammation has typically been associated with bacterial or viral infections, but it can also be induced by exposure to allergens and particulates like dust, chemicals and inhalable fibers that mimic proinflammatory stimuli, and can link inflammation to tumorigenesis. Smoking exemplifies such a non-infection, lifestyle-based inflammation, in which global epigenetic alterations, ranging from dysregulated histone and DNA methylation to aberrant microRNA expression patterns, can promote lung carcinogenesis (Sharma et al., 2010). Seiler et al. (2020) recently revealed that inflammation-induced modifications upset the balance of DNA methylation and demethylation in the lungs of nicotine-addicted mice, resulting in changes to histone acetylation levels and concomitant gene expression profiles that facilitate the development of lung cancer. Epigenetic modulations can also be actuated by hormonal treatments like sex steroids, which were demonstrated to change methylation levels and gene expression of various inflammatory signaling factors in prostate cancer patients (Wang et al., 2016).

### IL-6

Numerous inflammatory molecules can incite epigenetic disruptions, particularly in DNA methylation, which in turn promote various facets of cancer development in diverse cell types. IL-6 is one such example of a proinflammatory cytokine that orchestrates chronic inflammation, and has been connected to poor patient survival in different cancers (De Vita et al., 1998). NF-kB-mediated secretion of IL-6 from immune cells in cancer originating from colon inflammation appears to activate NF-kB and STAT3-dependent signaling in epithelial cells of the gastric mucosa, such as upregulation of DNA methyltransferase activity and associated methylome changes (Hartnett and Egan, 2012). Specifically, IL-6-directed increase in DNMT1 expression led to the hypermethylation and consequent repression of adhesion, apoptosis and tumor suppressor genes, thereby contributing to inflammation-linked colon tumorigenesis (Foran et al., 2010). In another study, inflammation caused by IL-6 in oral squamous cell carcinoma was responsible for reducing global methylation levels of LINE-1 retrotransposons, while increasing promoter methylation and concomitant silencing of select tumor suppressor genes (Gasche et al., 2011). Prior reports have also provided critical insights into the epigenetic mechanisms that govern the IL-6-induced generation of cancer stem cells (Drost and Agami, 2009; Iliopoulos et al., 2009; Iliopoulos et al., 2010), which are a subset of chemo-resistant tumor cells that drive cancer metastasis (Yu et al., 2012).

A well-established gene regulatory network that links IL-6-mediated chronic inflammation with cancer consists of two distinct but complementary feedback loops, one involving IL-6, NF-kB, Lin28 and let-7 miRNA, and the other comprising IL-6, NF-kB, STAT3, miR-181b-1, miR-21, CYLD and PTEN (Iliopoulos et al., 2009; Iliopoulos et al., 2010). In the former loop, activation of the Src oncogene *via* IL-6 secretion induces a proinflammatory output that is mediated by NF-kB, which leads to the increased expression of Lin28, an RNA binding factor that interacts with and impedes the expression of let-7 miRNA (Kumar et al., 2008). Loss of let-7, which usually targets IL-6, causes IL-6 accumulation, which then induces NF-kB, thereby creating a positive feedback circuit that sustains human breast cancer cells in a transformed state (Drost and Agami, 2009; Iliopoulos et al., 2009). As for the latter loop involving the STAT3 TF, which is induced by IL-6 that supports NF-kB in its active form, STAT3 triggers miR-181b-1 and miR-21 expression, which target the CYLD and PTEN tumor suppressor genes, respectively, resulting in the activation of NF-kB (Iliopoulos et al., 2010). Therefore, IL-6 works synergistically with the TFs NF-kB and STAT3, as well as multiple miRNAs, to set up dynamic regulatory feedback loops for perpetuating inflammatory cues that promote chronic inflammation and cancer.

### IL-1β

IL-1β is another potent proinflammatory cytokine that is not only abundantly expressed within the tumor microenvironment of several cancers, but is also a key contributor to various aspects of cancer development, including tumor growth, angiogenesis and metastasis (Elaraj et al., 2006; Voronov et al., 2007). In gastric cancer, IL-1β promotes DNA methyltransferase function *via* the synthesis of nitric oxide, resulting in promoter CpG island methylation-induced gene repression (Hmadcha et al., 1999). Similarly, IL-1β-mediated inflammatory signaling accounted for the promoter hypermethylation and gene silencing of E-cadherin, which is important for impeding cell migration and metastasis, based on a mouse model of gastric cancer (Huang et al., 2016). IL-1β has also been demonstrated to re-wire the DNA methylome of colon cancer cells by increasing DNMT3a and ablating DNMT3b expression, with minimal changes to DNMT1, leading to reduced CpG island methylation at the promoter regions of the IL-6 and IL-8 proinflammatory cytokine genes (Caradonna et al., 2018).

Further to the inflammation-mediated epigenetic changes at the primary tumor location, the interplay between inflammatory signaling and epigenetic mechanisms is also pertinent to cancer metastasis, especially during epithelial-to-mesenchymal transition (EMT), a trans-differentiation process by which transformed epithelial cells are reprogrammed to acquire mesenchymal features for invading and spreading to other sites of the body (Lopez-Novoa and Nieto, 2009; Suarez-Carmona et al., 2017). A case in point is the activation of the EMT program in IL-1β-induced non-small cell lung cancer (NSCLC) that facilitates epigenetic alterations at the E-cadherin gene promoter (Li R. et al., 2020). Mechanistically, acute exposure to IL-1β raises the expression level of a key EMT TF, SLUG, causing a decrease in active histone marks like H3K9ac and H3K4me3, while increasing inactive histone marks like H3K27me3. Chronic IL-1β exposure engenders greater accumulation of SLUG that induces *de novo* deposition of H3K9me2/3 and further enriches H3K27me3, collectively reinforcing E-cadherin gene repression during EMT memory (Li R. et al., 2020). Another related study revealed that IL-1β triggers oncogenic Lin28B expression by repressing miR-101, thereby dysregulating cellular proliferation and migration in inflammation-induced NSCLC (Wang et al., 2014).

### TGF-β

TGF-β is an anti-inflammatory cytokine that can activate the gene expression of DNA methyltransferases, which in turn alters the methylome of ovarian cancer cells during EMT (Cardenas et al., 2014). A similar function of TGF-β is recapitulated in breast cancer, whereby TGF-β robustly induces a suite of oncogenic EMT TFs like SNAIL, SLUG and TWIST1 to engage the EMT transcriptional program by upregulating mesenchymal cell-specific genes and antagonizing the expression of epithelial cell markers (Dong et al., 2012; Dong et al., 2013). Mechanistically, SNAIL-dependent repression consists of its interaction with the histone methyltransferases SUV39H1 and EHMT2 that collaborate to catalyze the deposition of the transcriptionally repressive histone modification H3K9me3, which is essential for recruiting DNA methyltransferases to carry out promoter methylation and stable silencing of target genes such as E-cadherin (Dong et al., 2012; Dong et al., 2013; Tam and Weinberg, 2013). TGF-β-induced EMT in breast cancer *via* the action of DNA and histone methyltransferases is also instrumental for the generation of cancer stem cells (Dong et al., 2012; Dong et al., 2013; David and Massague, 2018).

TGF-β signaling can trigger the expression of another epigenetic player, KDM6B, a histone demethylase that erases the silencing H3K27me3 mark to promote gene transcription, and this is crucial for the activation of SNAIL-induced EMT in both human and mouse mammary epithelial cells (Ramadoss et al., 2012). In support of this, Ramadoss et al. reported a dramatic elevation of KDM6B expression in metastatic breast cancer relative to healthy breast cells (Ramadoss et al., 2012). Additionally, stimulation of the EMT program by TGF-β in mammary epithelial cells leads to an increase in SIRT1 expression, which induces histone deacetylation and represses miR-200a expression (Eades et al., 2011). Because miR-200a targets SIRT1, both these epigenetic factors regulate each other via a negative feedback loop (Eades et al., 2011), and similar reciprocal feedback circuits have also been demonstrated in other studies between the ZEB family of EMT TFs and members of the miR-200 family that mutually regulate one another’s expression, thereby dynamically controlling the EMT transcriptional network (Shimono et al., 2009; Wellner et al., 2009).

Other noteworthy examples of epigenomic re-wiring driven by TGF-β-induced EMT include a widespread diminution of the silencing histone mark H3K9me2, and increase in the transcriptionally competent marks H3K4 and H3K36 trimethylation. These chromatin alterations rely on the LSD1 demethylase, as LSD1 depletion exerts adverse impacts on EMT-linked cancer cell metastasis and chemoresistance (McDonald et al., 2011). In addition to its role in TGF-β signaling, LSD1 can also participate in the NF-kB-mediated inflammatory pathway, as nuclear PKCα phosphorylates LSD1 to enable the binding and stabilization of NF-kB, suggesting that the PKCα-LSD1-NF-kB regulatory axis is important in the epigenetic control of EMT and its associated inflammatory phenotypes (Kim et al., 2018).

Finally, the dual crosstalk between inflammatory signaling and epigenetic modulations can set up a self-regulatory feedback circuit as a homeostatic mechanism to finetune the expression of inflammatory genes. This is elegantly illustrated in a seminal study by Foster et al. (2007), who showed that the robust activation of proinflammatory genes at the onset of LPS treatment was significantly muted upon subsequent stimulations. This was attributed in part to the maintenance of low histone H4 acetylation levels at the promoter regions of proinflammatory genes after the second round of LPS challenge, which reflects the dynamics of inflammatory gene activation and explains why macrophages appeared to lack sensitivity toward subsequent rounds of LPS induction (Foster et al., 2007).

In a different study, Cheng et al. (2013) discovered that canonical inflammatory genes like chemokines and adhesion factors were rapidly upregulated upon initial treatment with the proinflammatory cytokine TNFα, but their expression reduced over time despite continuous TNFα treatment. Yet, miR-146α/β expression displayed the opposite trend—higher induction at later compared to earlier timepoints of TNFα stimulation, which accounts for miR-146α/β activation coinciding with the downregulation of genes encoding adhesion factors, and that miR-146α/β served as a negative regulator of inflammatory signaling by targeting IRAK1, IRAK2 and TRAF6, thereby intricately controlling the ideal level of inflammatory output (Cheng et al., 2013). Taken together, inflammation-induced changes to the epigenome can efficiently feedback onto subsequent waves of inflammatory challenge to refine the overall kinetics of the inflammatory gene regulatory network, so as to avoid the deleterious outcome of excessive and uncontrolled inflammation.

## Epigenetic and Anti-inflammatory Therapies in Cancer

Given the closely intertwined nature between inflammatory signaling and epigenetic alterations, and how their dynamic bidirectional interaction augments oncogenesis, it is therefore not surprising that the administration of drug therapeutics targeting either or both pathways hold significant value in combating cancer. For instance, the immunosuppressive drug tocilizumab not only antagonizes IL-6-STAT3 inflammatory signaling, but also restrains the IL-6-STAT3-NF-kB epigenetic feedback axis, which heralds an exciting therapeutic prospect for triple-negative breast cancer patients (Alraouji et al., 2020).

Importantly, certain anti-inflammatory drugs are capable of functioning at the epigenetic level as well, such as non-steroidal anti-inflammatory drugs (NSAIDs) that can alleviate cancer progression by regulating the expression of HDACs. For example, the application of a commonly utilized NSAID, aspirin, in a mouse model of colitis-linked colon cancer, led to a decrease in the active histone H3K27ac levels and accompanying repression of the proinflammatory genes TNFα, IL-6 and inducible nitric oxide synthase (iNOS) (Guo et al., 2016). Aspirin also heightened the efficacy of another HDAC inhibitor drug, romidepsin, by boosting p21 expression, thereby hindering tumorigenesis in COX-1-positive ovarian cancer (Son et al., 2010). Additional support for NSAIDs in epigenetically mitigating cancer oncogenesis is documented in a recent report that long term treatment with ibuprofen correlates with a lower propensity to develop certain cancers (Shen et al., 2020). Specifically, ibuprofen not only suppressed numerous inflammation-associated stemness genes in breast, liver and lung cancer cells, but also decreased cancer cell metastasis and chemoresistance *via* the downregulation of HDAC and histone demethylase KDM6A/B both *in vitro* and *in vivo* (Shen et al., 2020).

Similarly, several epigenetic drugs possess the ability to counter inflammation. For instance, treatment with resveratrol and MS-275, a SIRT1 activator and a HDAC inhibitor, respectively, elicited anti-inflammatory properties by impeding microglia-macrophage activation in a mouse model of permanent brain ischemia (Mota et al., 2020). Another study revealed that administration of 5-azacytidine, a DNA methyltransferase inhibitor, and trichostatin A, another HDAC inhibitor, abrogated inflammation-dependent pyroptosis and apoptosis in acute lung injury *via* the repression of IL-1 and select caspase activities in bone-marrow-derived macrophages (Samanta et al., 2018). DNA methyltransferase inhibitors were similarly touted as a promising class of therapeutic candidates for tackling pancreatic cancer, as induction of NF-kB inflammatory signaling in pancreatic cancer stem cells requires DNA methylation of the promoter region of SOX9, a critical gene for cancer metastasis (Sun et al., 2013).

In the past decade, BET inhibitors, a prominent category of epigenetic drugs targeting the BET domain, which are bromodomain-containing proteins with well-established roles in gene regulation via histone modification and chromatin remodeling (Fujisawa and Filippakopoulos, 2017), have been successfully developed for various cancer therapies, including hematological tumors and the comparatively uncommon nuclear protein in testis (NUT) midline carcinomas (Filippakopoulos et al., 2010; Gallenkamp et al., 2014). Nicodeme et al. (2010) manufactured a synthetic histone mimic named I-BET that interferes with the binding of BET proteins to acetylated histones, resulting in the inhibition of chromatin complex formation necessary for inflammatory gene transcription in activated macrophages. This highlights the anti-inflammatory potential of synthetic compounds that specifically target proteins recognizing epigenetically modified histones in modulating physiological and pathological cell states.

Other documented examples of BET inhibitors include ABBV-075 and I-BET151, which were shown to exude apoptotic functions in multiple blood disorders like acute myeloid leukemia and non-Hodgkin’s lymphoma (Dawson et al., 2011; Bui et al., 2017). Notably, these epigenetic drugs also harbor anti-inflammatory characteristics, e.g., I-BET151 hampers the expression of the proinflammatory genes IL-1β and TNFα in rheumatoid arthritis synovial fibroblasts, leading to a decreased ability in recruiting immune cells and their lowered proliferative capacity (Klein et al., 2016). A recent report by Ullmann et al. (2021) demonstrated that treatment with the BET inhibitors I-BET151 and Ro 11–1,464 in cultured macrophages not only increases endogenous levels of the tumor suppressor protein CEBPD, but also downregulates key cytokine genes like CCL2 and IL-6, buttressing their anti-inflammatory functions. Furthermore, beyond the realm of drug therapeutics, natural dietary supplements like Vitamins C, D and E can also enact both anti-inflammatory and epigenetic effects (Saccone et al., 2015; Gerecke et al., 2018; Zappe et al., 2018; Yang et al., 2019).

## Conclusion

Epigenetic processes at various hierarchical levels of the genome take place in response to environmental stimuli, especially during infections and other inflammatory challenges, thereby modulating gene expression networks that govern cell identity and disease states. The aforementioned studies described here clearly illustrate the intimate connection between epigenetics and inflammation, and how they interact with each other through various feedback loops and regulatory axes, especially in the context of cancer. Owing to the reversible nature of epigenetic alterations and their susceptibility to inflammatory signaling from both internal and external environments, it is of paramount importance to decipher how these molecular mechanisms drive cancer initiation and progression. For example, prior studies have pinpointed the fundamental role of deleterious epigenetic modifications, particularly in DNA methylation profiles, in promoting inflammation-induced tumorigenesis (Chan et al., 2003; Maekita et al., 2006).

Importantly, the reversibility of epigenetics enables them to be harnessed as ideal cancer therapeutics to target the epigenetic changes within both the tumor core and microenvironment. High-throughput epigenomic and metabolomic approaches can be leveraged to elucidate a more thorough understanding of the repertoire of epigenetic and inflammation-related alterations in patient-derived cancer tissues, so that the appropriate treatments can be tailored to each patient. The combination of epigenetic drugs with anti-inflammatory roles, and vice versa, promise to offer much propitious prospects in long term palliative care and cancer therapy.
